# Comparison of topical estrogen and platelet-rich plasma injections in the treatment of postmenopausal vaginal atrophy

**DOI:** 10.3389/fmed.2025.1590078

**Published:** 2025-05-19

**Authors:** Ufuk Atlihan, Can Ata, Onur Yavuz, Huseyin Aytug Avsar, Selcuk Erkilinc, Tevfik Berk Bildaci

**Affiliations:** ^1^Department of Obstetrics and Gynecology, Merkezefendi Hospital, Manisa, Türkiye; ^2^Department of Obstetrics and Gynecology, Buca Seyfi Demirsoy Training and Research Hospital, İzmir Democracy University, İzmir, Türkiye; ^3^Department of Obstetrics and Gynecology, Dokuz Eylul University School of Medicine, İzmir, Türkiye; ^4^Department of Obstetrics and Gynecology, Tinaztepe University School of Medicine Galen Hospital, İzmir, Türkiye

**Keywords:** atrophic vaginitis, Female Sexual Function Index, platelet-rich plasma, topical estrogen, Vaginal Health Index

## Abstract

**Background:**

Platelet-rich plasma (PRP) is considered safe and is a low-cost, simple, natural, and minimally invasive method for vaginal rejuvenation. We aimed to compare the effects of hormonal treatment options and PRP use for postmenopausal vulvovaginal atrophy (VVA).

**Methods:**

From a total of 66 patients, topical estrogen treatment was administered to 36 patients, and PRP treatment was used on 30 patients who had previously received topical estrogen treatment without obtaining a response. To assess the impact of VVA and associated symptoms on the quality of life of patients, three different questionnaires, namely the Vaginal Health Index (VHI), Female Sexual Function Index (FSFI), and the Vulvovaginal Symptoms Questionnaire (VSQ), along with a Visual Analog Scale (VAS), were administered at 4-week intervals.

**Results:**

In the assessment at the 12th week, the FSFI and VSQ results obtained in patients treated with PRP showed a significant difference compared with those treated with topical estrogen (*p* = 0.004 and *p* < 0.001, respectively).

**Conclusion:**

PRP injections are a safe and effective minimally invasive monotherapy for postmenopausal VVA and, consequently, vulvovaginal rejuvenation. PRP injections are regarded as a promising method for the treatment of VVA in postmenopausal patients with contraindications to hormone therapy and improving hydration of the vaginal mucosa.

## 1 Background

Menopause is known to be a gradual, physiologic event associated with ovarian insufficiency, leading to significant effects on the lower genital tract due to hypoestrogenism (1, 2). With increasing life expectancy, postmenopausal vulvovaginal atrophy (VVA) has become more prominent in current medical practice, given its impact on quality of life, sexual function, and pelvic floor health (3, 4). Under hypoestrogenic conditions, the vaginal epithelium undergoes thinning, the barrier function is lost, vaginal folds decrease, tissue elasticity diminishes, and the secretion activity of Bartholin glands decreases, which results in vaginal mucosal traumatization and painful sensations (5). Symptoms accompanying vaginal dryness due to hypoestrogenism may include itching, burning, discharge, and dyspareunia. The frequency of VVA, the severity of pathologic changes, and the clinical course of the disease are dependent on the length of the postmenopausal period (6).

The main therapeutic goal in postmenopausal vaginal atrophy is the alleviation of symptoms. Low-dose vaginal estrogen therapy (ET) is the most widely used pharmacologic treatment for VVA and is also the most effective and safest option. They facilitate the renewal of the epithelium and vaginal flora, leading to the improvement of urogenital and sexual symptoms (6). During the use of low-dose vaginal ET, systemic estrogen absorption is minimal, and serum estradiol levels remain at postmenopausal levels (7–9).

In recent years, opinions and data on the use of platelet-rich plasma (PRP) in the treatment of postmenopausal vaginal atrophy have come into focus, becoming an actively discussed topic. PRP is considered safe and is a low-cost, simple, natural, and minimally invasive method for vaginal rejuvenation. Autologous growth factors, which are the essentials for PRP, include molecules such as vascular endothelial growth factor, epidermal growth factor, and platelet-derived growth factor (PDGF), which stimulate cell proliferation and cell differentiation. These molecules play a crucial role in reducing inflammation, stimulating collagen III synthesis, promoting angiogenesis, and ultimately contributing to tissue regeneration. PDGF has been reported to stimulate cell proliferation and participate in wound healing (10, 11). Studies on PRP use in VVA are limited in the literature (12, 13). Our study aimed to compare the effects of ET and PRP use for postmenopausal VVA.

## 2 Materials and methods

A total of 66 patients diagnosed with postmenopausal atrophic vaginitis who presented to our gynecology clinic between January 2019 and February 2022 were included in the study. Our research was designed as a retrospective observational study. The patients were divided into two groups based on the use of topical estrogen and PRP. Topical ET was administered to 36 patients, and PRP treatment was performed on 30 patients who had previously received topical ET without obtaining a response. Patients receiving ET were given a vaginal cream containing 0.625 mg per dose conjugated estrogen for 14 days, followed by single-dose applications every other night for the following 10 weeks. Conjugated topical estrogen was administered intravaginally using an applicator. The patients in the PRP group were those who received topical estrogen therapy before PRP application and did not respond to the treatment. In the treatment protocol of these patients, the patients were given a vaginal cream containing 0.625 mg conjugated estrogen every day for the first 14 days (2 weeks) and then received topical estrogen therapy every other night for the next 10 weeks. In other words, the patients applied topical estrogen one night and did not apply it the other night. The first 14 days (2 weeks) were planned as loading treatment and the other 10 weeks as maintenance treatment. The same protocol was applied to all patients. The patients selected for this group were included as a treatment option for patients who did not respond to topical estrogen treatment.

Initially, venous blood samples were drawn and centrifuged at 2,000 rpm for 10 min. The collected plasma was then subjected to centrifugation again in a plain tube at the same parameters. Platelet pellets and plasma samples were subsequently combined in a third tube. The entire process, including blood collection, processing, and preparation of PRP serum, and genital PRP application, took around 45 min. The PRP preparation process was performed by the same technician who had 5 years of experience in this field. A 4 mL PRP treatment was administered using 30-G needles, with 1 mL injected separately into each of the four vaginal walls. During the applications, injections were made into the anterior, posterior, and lateral walls of the vagina at approximately 1-cm intervals. Injections were applied to each wall, from distal to proximal. Injectors with a needle length of 13 mm were used. Applications were made after the 13 mm needle tip was completely inserted into the vaginal tissue at the steepest possible angle. The distal wall was reached by using a long insulin syringe. This procedure was preceded by the application of topical anesthetic cream. Sessions were repeated every 4 weeks for a total of three sessions. Minimal bleeding occurred at the injection sites after treatment. However, due to the local anesthesia applied, patients did not describe pain during the procedure. No pain symptoms were reported afterwards. Coitus was prohibited for the first week after the procedure. No additional reepithelializing cream application was required. To assess the impact of VVA and associated symptoms on the quality of life of patients, three different questionnaires, namely the Vaginal Health Index (VHI) (14), Female Sexual Function Index (FSFI) (15), and Vulvovaginal Symptoms Questionnaire (VSQ) (16), along with a Visual Analog Scale (VAS) (17), were administered at weeks 0, 4, 8, and 12. The VHI used a scale from 1 to 5 to analyze five components: elasticity, fluid volume, pH, epithelial integrity, and moisture. The minimum total score of 5 points indicates severe VVA, and the maximum total score of 25 points indicates the absence of clinical symptoms of VVA. The VAS scale ranges from 0 (complete absence of symptoms) to 10 (worst possible symptoms). Participants rated VVA symptoms (dyspareunia, dryness, or burning) on a scale from 0 to 10. The FSFI questionnaire, assessing six different domains—desire, arousal, lubrication, orgasm, satisfaction, and pain/discomfort used a scale from 0 (no sexual activity in the last 4 weeks) or 1 (very dissatisfied) to 5 (very satisfied) at weeks 0, 4, 8, and 12. Throughout the study, a full-scale score ranging from 2.0 (severe dysfunction) to 36.0 (no dysfunction) was employed to evaluate sexual function, considering that increased FSFI scores were associated with symptom improvement. An optimal cut-off score of 26, reported by Wiegel et al. (18), is used to distinguish women with and without current sexual dysfunction. The VSQ consists of 21 questions, using a scale ranging from 0 to 21 for evaluation.

The study included postmenopausal women aged between 45 and 70 years who were diagnosed as having VVA and not currently on any systemic estrogen therapy for any reason. Women with a history of breast or endometrial cancer, abnormal uterine bleeding, acute thrombophlebitis, or previous thromboembolic disorders related to estrogen use in the past 6 months before the study, and those with any contraindications to estrogen therapy were excluded from the study. The study was conducted in accordance with the principles of the Declaration of Helsinki. The study received approval from our hospital’s Ethics Committee (Number: 2023/168). Consent forms were obtained from the patients. Statistical analysis of the research data was conducted using the SPSS version 26 software package (IBM SPSS Statistics, IBM Corporation, Armonk, NY, United States). The Levene test was used to assess whether the study group exhibited equal variance and test the homogeneity of variance. Skewness and kurtosis in the statistical data were within the range of −1.5 to +1.5, indicating that the data conformed to normal distribution; therefore, paired sample *t*-tests were conducted for dependent groups. Kruskal–Wallis analysis was performed to evaluate the significance of the differences between group means. The results were evaluated at a significant level of *p* < 0.05 with a confidence interval of 95%.

## 3 Results

The mean ages of the volunteer participants, categorized into those using topical estrogen and those undergoing PRP treatment, were 57.17 ± 8.02 and 57.20 ± 7.75 years, respectively (range, 45–70 years) (*p* = 0.986). When considering the initial assessment data for all the tests used in the evaluation, a difference was observed only in the VSQ between the groups. In terms of other assessments during the baseline period, the groups were similar. In the assessment at the 12th week, the results obtained from the use of FSFI and VSQ in patients treated with PRP showed a significant difference compared with those treated with topical estrogen (*p* = 0.004 and *p* < 0.001, respectively) (Table 1).

**Table 1:** Comparison of baseline and endpoint data between groups.

Variables		Topical estrogen group (*n* = 36)	PRP group (*n* = 30)	*p*
		Mean ± SD		
Age		57.17 ± 8.02	57.20 ± 7.75	0.986
VHI scoring	Initial	12.58 ± 3.38	12.80 ± 3.43	0.798
	4th week	13.83 ± 2.94	14.16 ± 2.79	0.348
	8th week	14.66 ± 2.64	15.16 ± 2.16	0.279
	12th week	14.78 ± 2.43	15.67 ± 1.86	0.107
FSFI scoring	Initial	22.47 ± 1.03	22.77 ± 0.94	0.232
	4th week	23.25 ± 1.07	23.53 ± 0.73	0.342
	8th week	23.41 ± 0.99	23.96 ± 0.88	0.366
	12th week	23.50 ± 0.85	24.13 ± 0.86	0.004
VSQ scoring	Initial	13.86 ± 1.13	15.07 ± 0.91	<0.001
	4th week	13.08 ± 0.99	14.36 ± 0.61	<0.001
	8th week	12.80 ± 0.88	13.86 ± 0.34	<0.001
	12th week	12.64 ± 0.76	13.70 ± 0.47	<0.001
VAS scoring (dyspareunia)	Initial	6.97 ± 1.23	6.67 ± 1.37	0.344
	4th week	6.86 ± 1.06	6.46 ± 1.07	0.616
	8th week	6.75 ± 0.93	6.33 ± 0.92	0.632
	12th week	6.67 ± 0.89	6.27 ± 0.83	0.066
VAS scoring (dryness)	Initial	6.17 ± 1.25	6.27 ± 1.08	0.733
	4th week	6.11 ± 1.08	6.16 ± 0.87	0.812
	8th week	5.97 ± 0.98	6.15 ± 0.82	0.308
	12th week	5.97 ± 0.94	6.13 ± 0.82	0.466
VAS scoring (burning)	Initial	6.11 ± 1.09	6.17 ± 0.87	0.823
	4th week	6.02 ± 1.08	6.10 ± 0.75	0.784
	8th week	5.83 ± 0.84	6.00 ± 0.64	0.332
	12th week	5.75 ± 0.84	5.97 ± 0.61	0.245

Assessments were made using one-way ANOVA for group comparisons.

In participants receiving topical estrogen, statistically significant differences were observed between VAS measurements for 0–4 weeks, 0–8 weeks, and 0–12 weeks for dyspareunia (*p* = 0.044, *p* = 0.044, and *p* = 0.010). A statistically significant difference was also found between VAS measurements for 0–8 weeks and 0–12 weeks for burning sensation (*p* = 0.010 and *p* = 0.002, respectively). No statistically significant difference was observed in VAS measurements for dryness sensation among participants undergoing topical estrogen treatment (*p* > 0.05).

For participants undergoing PRP treatment, statistically significant differences were observed between VAS measurements for 0–8 weeks and 0–12 weeks for dyspareunia (*p* = 0.010 and *p* = 0.005, respectively). No statistically significant difference was observed in VAS measurements for dryness sensation among participants undergoing PRP treatment (*p* > 0.05). A significant difference was found between VAS measurements for 0–12 weeks for burning sensation in participants undergoing PRP treatment (*p* = 0.031) (Table 2).

**Table 2:** Week-to-week analysis of differences compared with the initial results between the two groups.

Variables		Topical Estrogen (*n* = 36) mean ± SD	*p*	PRP (*n* = 30) mean ± SD	*p*
VHI scoring	4th week	1.25 ± 0.65	<0.001	1.37 ± 0.72	<0.001
	8th week	2.08 ± 0.97	<0.001	2.37 ± 1.45	<0.001
	12th week	2.19 ± 1.28	<0.001	2.87 ± 1.98	<0.001
FSFI scoring	4th week	0.78 ± 1.05	<0.001	0.77 ± 0.97	<0.001
	8th week	0.94 ± 1.09	<0.001	1.20 ± 1.10	<0.001
	12th week	1.03 ± 1.08	<0.001	1.37 ± 1.13	<0.001
VSQ scoring	4th week	−0.78 ± 0.76	<0.001	−0.70 ± 0.75	<0.001
	8th week	−1.06 ± 0.95	<0.001	−1.20 ± 0.96	<0.001
	12th week	−1.22 ± 0.96	<0.001	−1.37 ± 1.07	<0.001
VAS scoring (dyspareunia)	4th week	−0.11 ± 0.32	0.044	−0.20 ± 0.61	0.083
	8th week	−0.22 ± 0.64	0.042	−0.33 ± 0.66	0.010
	12th week	−0.31 ± 0.67	0.010	−0.40 ± 0.72	0.005
VAS scoring (dryness)	4th week	−0.56 ± 0.33	0.324	−0.10 ± 0.40	0.184
	8th week	−0.19 ± 0.58	0.051	−0.13 ± 0.43	0.103
	12th week	−0.19 ± 0.56	0.052	−0.13 ± 0.45	0.105
VAS scoring (burning)	4th week	−0.08 ± 0.28	0.083	−0.07 ± 0.25	0.161
	8th week	−0.28 ± 0.62	0.010	−0.17 ± 0.46	0.057
	12th week	−0.36 ± 0.64	0.002	−0.20 ± 0.48	0.031

The assessment involved comparing the initial questionnaire scoring results for each successive week of the study, stratified by the administration of either topical estrogen or PRP. The analysis was performed using the paired-samples test.

## 4 Discussion

In our study, we evaluated the mean differences in responses to questionnaires administered at 0, 4, 8, and 12 weeks following topical estrogen and PRP administration to monitor outcomes. Previously, in the REVIVE study, reported symptoms of vaginal atrophy in postmenopausal women included vaginal dryness (55%), dyspareunia (44%), vaginal irritation (37%), vaginal sensitivity (17%), bleeding during sexual intercourse (8%), and pain during exercise (2%) (19). In a separate study, it was stated that vaginal dryness and dyspareunia were the most prevalent symptoms, and itching, burning, vaginal discharge, a sensation of fullness, and coital bleeding might manifest as the condition progresses (20). In a study involving postmenopausal women aged 50–79 years, it was observed that 52% continued their sexual life, whereas in studies with women aged 70–79 years, this rate decreased to 22% (21). Looking at the frequency of sexual symptoms experienced by postmenopausal women in the literature, Liu and Eden (22) found the rate as 41%, and Chen et al. (23) reported a rate of 49.3%. In our study, we used a variety of questionnaires to address the different types of symptoms mentioned earlier in patients with VVA.

There was noted improvement in the VAS scores for dyspareunia between the 0–4th, 0–8th, and 0–12th weeks, as well as in the VAS scores for burning sensation between the 0–8th and 0–12th weeks, among the group using topical ET. In two similar studies, the use of low-dose vaginal estrogen tablets was shown to be effective in reducing postmenopausal urogenital symptoms and reducing postmenopausal dyspareunia (24, 25). FSFI scores and VHI scores consistently demonstrate symptom alleviation across all weeks of measurement, similar to findings from two other studies that also used the FSFI and VHI. These studies both indicated significant improvement in the respective scores following a 12-week topical estrogen therapy regimen (26, 27).

In two separate studies using the VHI, VSQ, and FSFI, significant improvements in scores were achieved in patients treated with PRP during their 1-month follow-up assessments (28, 29). In our study, participants who received PRP exhibited significant improvement in FSFI, VSQ, and VHI measurements throughout each week of the study, including the assessments conducted from week 0 to week 12. Hersant et al. (30) observed improvement in clinical symptoms such as vaginal dryness and dyspareunia in all participants in their study. In our study, participants who received PRP showed significant improvement in VAS scores for dyspareunia and burning sensation measurements from week 0 to week 12, but no significant improvement was observed for dryness sensation measurements. To date, including our study, no adverse effects have been reported in the literature regarding vaginal PRP applications. This is explained by the fact that the content of PRP is obtained from the patient’s own plasma.

There are various treatment methods described in the literature for postmenopausal vaginal atrophy. According to generally accepted international standards, the use of lubricants and moisturizers are the first-line recommendations for the treatment of mild and moderate VVA symptoms; non-hormonal vaginal lubricants that should be used before intercourse, and long-term effective vaginal moisturizers that are used regularly (several times a week) (31). However, in the study conducted by Caruso et al. (32), no significant difference was found in VHI and FSFI scores at the end of treatment in postmenopausal women using lubricants and moisturizers.

Topical anesthetic gels can be used for women with postmenopausal VVA symptoms. In the study conducted by Morin et al. (33), no significant improvement was found in VAS (dyspareunia) and FSFI scores after topical lidocaine treatment in patients with dyspareunia symptoms. Ospemifene acts as a selective estrogen receptor modulator for the treatment of VVA and dyspareunia. Di Donato et al. (34) revealed that treatment with ospemifene 60 mg/day was associated with significant improvement in the morphologic and physiologic characteristics of the vaginal mucosa, which are associated with symptoms associated with postmenopausal VVA. In the study by Archer et al. (35), a significant improvement was found in the dyspareunia symptoms and FSFI scores of patients using ospemifene. However, in another study, a statistical increase in endometrial thickness was detected both at the 12th week and the 52nd week after ospemifene treatment (36). In studies based on ospemifene, the possibility of triggering an increase in endometrial thickness is thought-provoking, despite its positive effects on postmenopausal VVA symptoms. In our study, we think that the absence of systemic adverse effects related to PRP may provide an advantage over ospemifene treatment in terms of safety.

Prasterone contains dehydroepiandrosterone (DHEA), which is a precursor of androgen and estrogen. Daily intravaginal application of 0.50% (6.5 mg) prasterone has clinically positive effects on dyspareunia and vaginal dryness (37). In the study by Bouchard et al. (38), a statistically significant increase in FSFI scores and a decrease in dyspareunia symptoms were found in women treated with prasterone compared with the beginning of treatment. However, in the study conducted by Barton et al. (39), no significant difference was found between the groups treated with prasterone and plain moisturizer in terms of changes in dryness or dyspareunia. In the case of vaginal treatment, levels of aromatase (the enzyme that converts androgen to estrogen) expressed in the endometrium appear to be minimal, indicating a low risk of endometrial hyperplasia or cancer. Short-term supplementation studies have shown no significant effects on the endometrium (40). However, there are limited data on the long-term safety of vaginal DHEA, particularly concerning the risk of cardiovascular disease and breast cancer. Prasterone has not been studied in survivors of breast cancer, and its use is contraindicated in patients with current or past breast cancer (41). Due to the lack of clinical efficacy and long-term safety data, the Endocrine Society recommends against widespread long-term use of DHEA in women (42). We think that the absence of systemic adverse effects related to PRP in our study may provide an advantage over prasterone treatment in terms of safety.

Fractional CO_2_ laser treatment for vulvovaginal atrophy is a safe and effective procedure in well-selected patients. In the study by Di Donato et al. (43), CO_2_ laser treatment was performed on patients with postmenopausal VVA symptoms. The mean overall pain score of all patients was evaluated on a 7-point Likert scale. A statistically significant decrease was found in the patients’ pain scores between the 1st and 3rd treatments. D’Oria et al. (44) evaluated the effectiveness of fractional CO_2_ laser use in the treatment of postmenopausal VVA in patients with gynecologic cancer. Fractional CO_2_ laser has been shown to improve clinical symptoms and sexual function in terms of VHI and FSFI scores. Fractional CO_2_ laser use in the treatment of VVA seems a safe therapeutic option. CO_2_ laser therapy has shown results comparable to PRP injections in patients with gynecologic cancer. Further studies, potentially involving combined therapies, are necessary to assess whether better outcomes can be achieved for this patient group, which lacks estrogen. To our knowledge, no other studies have compared the two methods for treating VVA. When comparing the effectiveness of topical estrogen and PRP injections, it is noteworthy that although there was no significant difference in initial FSFI and VHI scores, a significant difference was observed in favor of PRP at the end of the 12th week.

All injection sessions were generally well tolerated, and no acute adverse effects were reported at the injection sites. However, even though very thin needles were used, patients described minimal pain during needle insertion, especially in the distal part of the vagina. Minimal bleeding occurred at the injection sites in the first 24 h after the procedure. We believe that our study contributes to the literature by comparing the efficacy of these two treatment methods.

The strength of our study lies in the frequency of application and the duration of treatment, which surpasses that of other studies in the literature, many of which have shorter treatment durations. However, the limitations of our study include its relatively small sample size and the lack of investigation into the partner’s sexual dysfunction as a potential cause of female sexual dysfunction. Although we preferred to use the Vaginal Health Index in this study, not using the vaginal maturation index as an additional evaluation criterion is considered another limitation of the study.

## 5 Conclusion

PRP injections are a safe and effective minimally invasive monotherapy for postmenopausal VVA. PRP injections are regarded as a promising method for the treatment of VVA in postmenopausal patients with contraindications to hormone therapy and improving hydration of the vaginal mucosa. Larger randomized studies are necessary to comprehensively address the outcomes of primary PRP treatment for VVA.

## Data Availability

The original contributions presented in the study are included in the article/supplementary material, further inquiries can be directed to the corresponding author.
